# LRRC8A promotes the initial development of oxaliplatin resistance in colon cancer cells

**DOI:** 10.1016/j.heliyon.2023.e16872

**Published:** 2023-06-01

**Authors:** Haifeng Zhang, Zhenghui Jing, Rong Liu, Yassin Shada, Sindhwani Shria, Shiyu Cui, Yuhua Ren, Yuan Wei, Liangming Li, Shuang Peng

**Affiliations:** a Department of Pathology of Basic Medicine College, Xi'an Jiaotong University, Xi'an 710061, China; b Institute of Genetics and Developmental Biology of Translational Medicine Institute, Xi'an Jiaotong University, Xi'an 710049, Shannxi, China; c Key Laboratory of Sports Technique, Tactics and Physical Function of General Administration of Sport of China, Scientific Research Center, Guangzhou Sport University, Guangzhou 510500, China; d School of Sport and Health Sciences, Guangzhou Sport University, Guangzhou 510500, China

**Keywords:** Colon cancer, VRAC, LRRC8A, Drug resistance, Oxaliplatin

## Abstract

Leucine-rich repeat-containing 8 A (LRRC8A) is an essential component of the volume-regulated anion channel (VRAC), which plays a vital role in cell proliferation, migration, apoptosis, and drug resistance. In this study, we investigated the effects of LRRC8A on oxaliplatin resistance in colon cancer cells. The cell viability was measured after oxaliplatin treatment with cell counting kit-8 (CCK8) assay. RNA sequencing was used to analyze the differentially expressed genes (DEGs) between HCT116 and oxaliplatin-resistant HCT116 cell line (R-Oxa) cells. CCK8 assay and apoptosis assay indicated that R-Oxa cells significantly promoted drug resistance to oxaliplatin compared with native HCT116 cells. R-Oxa cells, deprived of oxaliplatin treatment for over six months (R-Oxa^dep^), maintained a similar resistant property as R-Oxa cells. The LRRC8A mRNA and protein expression were markedly increased in both R-Oxa and R-Oxa^dep^ cells. Regulation of LRRC8A expression affected the resistance to oxaliplatin in native HCT116 cells, but not R-Oxa cells. Furthermore, The transcriptional regulation of genes in the platinum drug resistance pathway may contribute to the maintenance of oxaliplatin resistance in colon cancer cells. In conclusion, we propose that LRRC8A promotes the acquisition rather than the maintenance of oxaliplatin resistance in colon cancer cells.

## 1 Introduction

According to the international agency for research on cancer (IRAC) statistics, colorectal cancer (CRC) is the second cause of cancer-related mortality worldwide, responsible for 0.93 million deaths globally in 2020 [1]. Since cisplatin was approved for anti-cancer therapies in 1978, platinum (Pt) compounds have been one of the most widely used classes of anticancer drugs [2]. Oxaliplatin, a third-generation platinum drug, is used as first-line chemotherapy in CRC. However, emerging drug resistance to oxaliplatin in colon cancer patients severely affects the therapeutic efficacy, resulting in the failure of chemotherapy.

In recent years, it has become increasingly clear that the regulation of ion channels and transporters' expression is an important mechanism in the development of drug resistance. Volume-regulated anion channel (VRAC) formed by a cell type-specific or tissue-specific subunit component was suggested to be strongly associated with enhanced cancer cell growth and metastasis [3]. It has been confirmed that cell volume regulation conducted by VRAC played a vital role in cell proliferation, migration, death, and multidrug resistance of cancer cells [[4], [5], [6], [7]]. Leucine-rich repeat-containing 8 (LRRC8) family proteins were identified as the main molecular components of VRAC, which was formed by Leucine-rich repeat-containing 8 A (LRRC8A) with at least one of LRRC8B/C/D/E as a regulatory subunit [8,9].

Several studies have reported the potential role of LRRC8A in drug resistance of different cancers, but these observations have not been consistent. Downregulation of LRRC8A promoted cisplatin resistance in human ovarian A2780 cancer cells and alveolar A549 adenocarcinoma cells [[10], [11], [12]]. Moreover, it enhanced resistance to carboplatin and cisplatin rather than oxaliplatin in HPA1 cells [13]. Overexpression of LRRC8A potentiated temozolomide sensitivity in glioma U87/R cells [14]. However, it was found that disturbing LRRC8A expression increased the sensitivity of glioblastoma to temozolomide and carmustine [15]. Furthermore, it was demonstrated that alteration of LRRC8A expression could not affect the response of lung adenocarcinoma A24 cells to cisplatin [16]. These findings indicated that the inconsistent effects of LRRC8A on drug resistance may be determined by a variety of factors, such as cell type and drug properties.

The role of LRRC8A in oxaliplatin resistance in colon cancer was still not elucidated clearly. In the present study, we successfully established oxaliplatin-resistant HCT116 cells (R-Oxa) by the drug-induced method, and the effect of LRRC8A on oxaliplatin resistance was investigated in colon cancer cells.

## 2 Materials and methods

### 2.1 Cell culture and infection

The native human colon cancer HCT116 cells were purchased and verified according to short tandem repeat (STR) standards by Genechem company (Shanghai, China). The native and drug-modified HCT116 cells were cultured in Roswell Park Memorial Institute (RPMI) 1640 (#C11875500BT, Gibco, USA) medium containing 10% fetal bovine serum (#10270-106, Gibco, Germany), 100 IU/ml penicillin, and 100 μg/ml streptomycin (#15140-122, Gibco, USA) and maintained as a monolayer in 25 cm^2^ tissue-culture flasks at 37 °C in a humidified atmosphere with 5% CO_2_ and 95% O_2_. The cells were trypsinized and subcultured (#25200-056, Gibco, Canada) every 2 days.

The sequence of specific short hairpin RNA (shRNA) for LRRC8A was 5′-CCGGACCAAGCTCATCGTCCTCAACCTCGAGGTTGAGGACGATGAGCTTGGTTTTTTG-3′, which was chemically synthesized and inserted into the pGLVH1 lentivirus shRNA vector carrying the GFP gene, and then packaged as infectious lentivirus by GenePharma (Shanghai, China), as previously reported [3]. The infectious lentivirus for over-expressing LRRC8A was purchased from Genechem (Shanghai, China). The coding sequence (CDS, NM_001127244) frame of LRRC8A was cloned in the GV260 vector carrying the luciferase gene.

For virus infection, colon cancer cells were plated in 12-well plates and grown to 30–40% confluency. Then lentivirus droplets [multiplicity of infection (MOI = 5)] were added into the cell culture medium. After 12 h of incubation at 37 °C, the medium was replaced with fresh media. Infected cells were observed under the fluorescence microscope (Olympus BX51, Tokyo, Japan) or the molecular imaging system (Xenogen, Alameda, CA, USA) and then used for downstream experiments.

### 2.2 Generation of oxaliplatin-resistant HCT116 cells (R-Oxa), recovering and cell viability assay

Oxaliplatin powder (#HY-17371, MCE, USA) was dissolved in sterile water and stored at a concentration of 5 mg/ml at −80 °C. The flowchart to generate the oxaliplatin-resistant colon cancer cells was shown in Fig. 1A. The native HCT116 cells were gradually exposed to the increasing concentrations of oxaliplatin from 1 μM to 6 μM, as follows: during the 1st and 2nd week, once a week for 24 h incubation with an initial oxaliplatin concentration (1 μM), then the sustaining cells at least 80% confluent were passaged. In the following five weeks, the concentrations of oxaliplatin increased from 2 μM to a peak concentration of 6 μM. Then the peak concentration was maintained once a week for five weeks. The resistance was finally examined by cell counting kit-8 (CCK8) assay. The oxaliplatin resistant cells were preserved with the administration of 6 μM oxaliplatin weekly and frozen in a liquid nitrogen container.

**Fig. 1:**
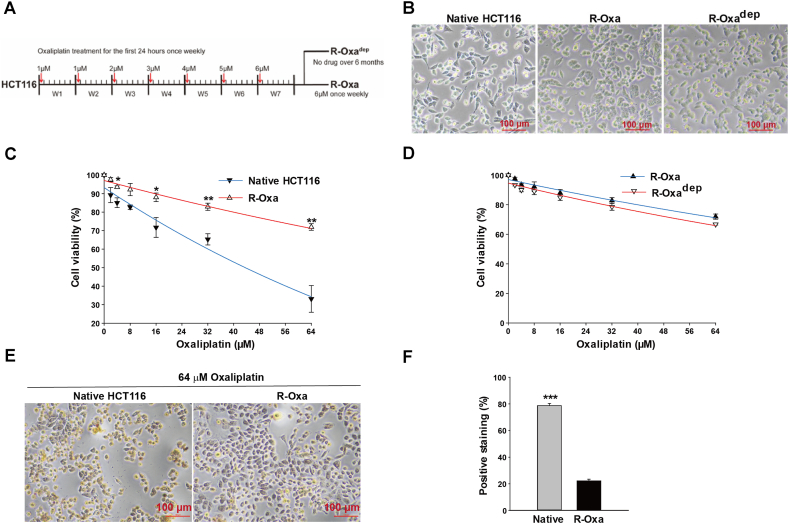
R-Oxa cells were established successfully with a drug-induced method. (A) The flowchart for establishing R-Oxa and R-Oxa^dep^ cells. (B) Representative images of native HCT116, R-Oxa, and R-Oxa^dep^ cells. (C) The cell viability of R-Oxa cells was significantly elevated compared to native HCT116 cells in the culture medium containing different concentrations of oxaliplatin. (D) R-Oxa^dep^ cells had a similar resistance characteristic to R-Oxa cells. The cell viability of R-Oxa and R-Oxa^dep^ cells was determined after incubation with the following concentrations of oxaliplatin (2, 4, 8,16, 32, 64 μM) for 24 h. (E) TUNEL staining was used to evaluate cell apoptosis in native HCT116 and R-Oxa cells treated with 64 μM oxaliplatin for 24 h. (F) Statistics of TUNEL positive staining cells in native HCT116 and R-Oxa cells treated with 64 μM oxaliplatin for 24 h. n = 3; *P < 0.05, **P < 0.01, ***P < 0.001.

To observe the stability of oxaliplatin resistance, R-Oxa cells were deprived of the weekly oxaliplatin treatment for over six months and R-Oxa^dep^ cells were obtained. Cell viability was analyzed by CCK8 assay (#CK04, Dojindo Laboratories, Japan). 1.5 × 10^4^ cells in the volume of 100 μl were seeded in 96-well plates. After incubation for 12 h at 37 °C, a variety of oxaliplatin solutions with concentrations of 2 μM, 4 μM, 8 μM, 16 μM, 32 μM, and 64 μM (or 5 μM, 15 μM, 30 μM, 60 μM, and 120 μM) were added in triplicate and incubated for 24 h. Then, 10 μl CCK-8 solution was added to every well. After 4 h of incubation, the absorbance at 450 nm was measured using a Multiskan FC microplate reader (Thermo Fisher Scientific, Waltham, MA, USA). The viability was calculated by the formula:

cellviability=(experimentalgroup−blankgroup)/(controlgroup−blankgroup)×100%

(blank group: culture medium; control group: cells without oxaliplatin incubation).

### 2.3 Cell apoptosis by TUNEL staining

Apoptosis assay of native HCT116 and R-Oxa cells was performed by colorimetric TUNEL apoptosis assay kit (C1091, Beyotime, China). Briefly, 1 × 10^5^ cells were seeded in 48 well-plates for 12 h, then cultured in a medium containing 64 μM oxaliplatin for 24 h. Cells were sequentially fixed with 4% paraformaldehyde for 30 min, permeabilized with 0.3% Triton X-100 for 5 min, and incubated with 0.3% H_2_O_2_ for 20 min to inactivate the endogenous peroxidases. After washing 3 times with PBS, the cells were incubated with 50 μl Biotin-dUTP working solutions (per well) containing 5 μl terminal deoxynucleotidyl transferase for 1 h at 37 °C. After termination for 10 min, cells were incubated with 50 μl streptavidin-HRP for 30 min at room temperature and washed 3 times with PBS. Then, cells were incubated with 200 μl diaminobenzidine (DAB) solution (per well) for 5 min and photographed with an inverted microscope (CKX53SF, OLYMPUS, Japan). The percentage of positively staining cells was calculated by ImageJ software (ImageJ, v1.8.0, USA).

### 2.4 Reverse transcription quantitative polymerase chain reaction (RT-qPCR)

Total RNA was extracted according to the specification of the Trizol reagent (#R4801, Magen, China) and quantified using the nanodrop instrument (Denovix, USA). 1 μg RNA was reverse-transcribed in a 20 μl reaction system using a cDNA Synthesis SuperMix (#1137ES60, Yeasen, China). RT-qPCR was performed using the BioRad CFX real-time system (CA, USA) with LRRC8A and GAPDH specific primers (#11201ES03, Yeasen, China) and the steps were: pre degeneration at 95 °C for 5 min; denaturation at 95 °C for 10 s, annealing and extension at 60 °C for 30 s for 40 cycles. The 2^−ΔΔCT^ method was used to calculate relative gene expression.

The sequences of primers for LRRC8A and GAPDH were designed as follows: LRRC8A forward (5′- CGGCCTCCTGCAGAACCTCC-3′), LRRC8A reverse (5′-GCTTCCGGCACTGGAAGAGC-3′), GAPDH forward (5′-TGACTTCAACAGCGACACCCA-3′), GAPDH reverse (5′-CACCCTGTTGCTGTAGCCAAA-3′).

### 2.5 Western blot analysis

Cells were harvested when they were grown to 80% confluency and lysed with radioimmunoprecipitation assay (RIPA) buffer (#P0013, Beyotime, China). The extracted proteins were quantified by bicinchoninic acid (BCA) protein assay (#20201ES76, Yeasen, China). After all protein samples were adjusted to the same concentration, proteins were mixed with the 5× protein loading buffer (PE015, ZHONGHUIHECAI, China) and boiled for 10 min to denature. 20 μg proteins were loaded into each lane, separated by 10% SDS-PAGE gel, and transferred onto a polyvinylidene difluoride (PVDF) membrane (#ISEQ00010, Millipore, USA). The membrane was blocked with 5% nonfat milk for 1 h at room temperature and immersed in the primary diluted antibody solution overnight at 4 °C (1:1000 for LRRC8A Rabbit Ab, #24979S; 1:1000 for β-Actin Mouse mAb, #3700S, CST, USA). After washing out the nonspecific conjugated proteins with TBST solution, the membrane was incubated in the solution containing the horseradish peroxidase-conjugated antibody (1:5000 for anti-Mouse IgG, #SA00001-1, Proteintech, China; 1:5000 for anti-Rabbit IgG, #RS0002, Immunoway, USA) for 1 h at room temperature. Finally, the membrane was rinsed adequately with TBST solution, and protein bands were detected using a commercially available enhanced chemiluminescent horseradish peroxidase (HRP) substrate kit (#WBKLS0100, Millipore, USA). Images were captured by the chemiluminescent Imaging and Analysis System (ChampChemi 610, China) and protein expression was analyzed with the ImageJ software (ImageJ, v1.8.0, USA).

### 2.6 Liquid chromatography/mass spectrometry

Protein gel identification was performed by liquid chromatography/mass spectrometry in MONITOR HELIX (shanghai, China). Briefly, the SDS-PAGE gel of the corresponding location was cut, reduced, alkylated, and digested into peptides with trypsin (10 ng/μl). 2 μl of the peptide solution was separated using a Phenomenex aqua C18 column with a 2 μm particle size in a 200-mm length 75 μm internal diameter, 100 Å pore size on UltiMate 3000 HPLC unit (UltiMate 3000, Dionex, USA) and identified using Q Exactive HF hybrid quadrupole-orbitrap mass spectrometer (Q Exactive HF, Thermo Fisher, USA). Raw data were analyzed with MaxQuant software (version 1.6.0.16) and screened according to FDR <0.01. High-confidence peptides were blasted with the UniProt human protein database (https://www.uniprot.org/) for protein identification.

### 2.7 RNA sequencing

Briefly, the total RNAs of native and R-Oxa HCT116 cells (biological quadruplicate) were extracted and checked for RNA-Seq loading and quality control. Then the enriched mRNA was fragmented into approximately 200 nt RNA inserts, which were used to synthesize the first-strand cDNA and the second cDNA. The double-stranded cDNA was end-repaired/dA-tailed and ligated to the adaptor. The suitable fragments were isolated by Agencourt AMPure XP beads (Beckman Coulter, Inc.), and enriched by PCR amplification. Finally, the constructed cDNA libraries were sequenced using an Illumina HiSeq™ sequencing platform.

Excluding the genes with a COUNT value of less than 5, differentially expressed genes (DEGs) between the groups of native and R-Oxa HCT116 cells were analyzed based on the edgeR package. Genes with *P*-values less than 0.05 and absolute fold changes (FC) of more than 1.2 were chosen for subsequent analyses. The annotation and KEGG pathway analysis of DEGs were performed on the BMKCloud (www.biocloud.net) platform.

### 2.8 Statistical analysis

Results were presented as mean ± standard error. Comparisons between groups were performed by an independent two-tailed Student's t-test or one-way analysis of variance followed by the Student-Newman-Keuls method for multiple comparisons. All analyses were performed in Sigmaplot software (v12.5, Systat Software Inc. USA) and *P* < 0.05 was considered to be statistically significant.

## 3 Results

### 3.1 Human colon cancer HCT116 cells acquired resistance to oxaliplatin after being exposed to increasing concentrations of oxaliplatin

R-Oxa cells were cultivated from the parental HCT116 cells, which were gradually treated with the concentrations of oxaliplatin from 1 μM to 6 μM for over three months (Fig. 1A). Compared to the native cells, there were fewer sharp cellular protrusions for the oxaliplatin-treated cells (Fig. 1B). Then the sensitivities of parental and exposed cells to oxaliplatin were measured by CCK-8 assay. As illustrated in Fig. 1C, in cell culture solutions containing 16, 32, and 64 μM oxaliplatin, cell viabilities of R-Oxa cells were much higher than those of native HCT116 cells (n = 3, *P* < 0.05). The R-Oxa cells being bathed with 64 μM oxaliplatin for 24 h had surprising viability of about 72%, suggesting that the exposed cells have acquired resistance to oxaliplatin. Half maximal inhibitory concentration (IC_50_) of oxaliplatin for native HCT116 cells was calculated as 43.82 μM. It is not observed that the viability of the R-Oxa cell was reduced by half at the maximal concentration of oxaliplatin, for which IC_50_ was theoretically calculated as 135.39 μM, far more than the clinical medication concentration.

We then further investigated whether the acquired resistance to oxaliplatin in R-Oxa cells would be stable. R-Oxa cells were deprived of the weekly oxaliplatin treatment for over six months and then R-Oxa^dep^ cells were obtained. As shown in Fig. 1D, the dose-effect curves for R-Oxa and R-Oxa^dep^ cells nearly overlapped. At the concentration of 64 μM oxaliplatin, the viabilities of R-Oxa and R-Oxa^dep^ cells were not different, but much higher when compared to the native HCT116 cells (n = 3, *P* < 0.01). IC_50_ of oxaliplatin for R-Oxa^dep^ cells was theoretically computed as 112.02 μM, being similar to that for R-Oxa cells, which suggested that R-Oxa cells and R-Oxa^dep^ cells have developed a stable resistance to oxaliplatin. Furthermore, terminal deoxynucleotidyl transferase (TdT)-mediated dUTP Nick-End Labeling (TUNEL) was used to evaluate the cell apoptosis after oxaliplatin treatment. As shown in Fig. 1E and F, native HCT116 cells treated with 64 μM oxaliplatin exhibited a significant increase in apoptosis than R-Oxa cells (n = 3, *P* < 0.001).

### 3.2 LRRC8A proteins were highly expressed in R-Oxa and R-Oxa^dep^ cells

Increasing evidence indicated LRRC8A is involved in chemotherapy drug resistance of multiple cancer cells, but this is not consistent. Here, we first examined whether the expression of LRRC8A was altered in R-Oxa and R-Oxa^dep^ HCT116 cells. Melting curves of the amplification products for GAPDH and LRRC8A processed a peak that confirmed the specificity of primers (Fig. 2A). LRRC8A mRNA and protein were quantitatively detected by qPCR and Western blot, respectively. The mRNA level of LRRC8A in R-Oxa cells was 1.97 ± 0.09 folds, higher than that in the native HCT116 cells (n = 3, *P* < 0.001, Fig. 2B). Similarly, LRRC8A protein expression in R-Oxa cells was significantly increased, which was 2.16 ± 0.35 folds of native HCT116 cells (n = 3, *P* < 0.01, Fig. 2C and D). In R-Oxa^dep^ HCT116 cells, mRNA and protein levels of LRRC8A were increased to 1.53 ± 0.10 (n = 3, *P* < 0.001, Figs. 2E) and 1.47 ± 0.12 folds, respectively (n = 4, *P* < 0.05, Fig. 2F and G). Compared to R-Oxa cells, R-Oxa^dep^ HCT116 cells had a 20%–30% reduction of LRRC8A protein expression after being chronically deprived of oxaliplatin. Taken together, the maintenance of strong LRRC8A expression may be conducted by the drug stimulation, suggesting that LRRC8A may be an important factor in the acquisition of oxaliplatin resistance for colon cancer cells.

**Fig. 2:**
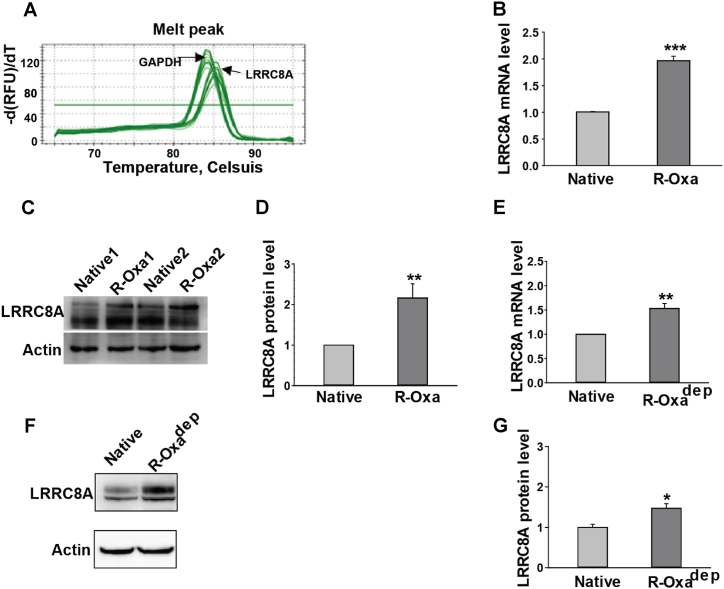
The expressions of LRRC8A were upregulated in R-Oxa and R-Oxa^dep^ cells. The transcriptional and translational levels of LRRC8A were detected by qPCR and Western blot, respectively. (A) Melting curves of the amplification products for GAPDH and LRRC8A. (B and E) The statistics show the mRNA levels of LRRC8A were increased in R-Oxa and R-Oxa^dep^ cells. (C and D) The protein expression of LRRC8A in R-Oxa cells was higher than that in native HCT116 cells (Supplementary Fig. 1). (F and G) The protein expression of LRRC8A in R-Oxa^dep^ cells was higher than that in native HCT116 cells (Supplementary Fig. 2). n = 3–4; *P < 0.05, **P < 0.01, ***P < 0.001.

### 3.3 Regulation of LRRC8A expression significantly altered resistance to oxaliplatin in native HCT116 cells

LRRC8A proteins were elevated during the chronic exposure to oxaliplatin, but it's not yet distinguished if the changed LRRC8A was an accompanied or indispensable event in the process of acquiring resistance to oxaliplatin in colon cancer cells. Hence, the sensitivity to oxaliplatin was evaluated when LRRC8A proteins were overexpressed or downregulated in the native HCT116 cells.

After HCT116 cells were infected with lentivirus packaging the over-expressing vectors of LRRC8A or scramble vectors for 72 h, the infected cells indicated powerful green fluorescence under the molecular imaging system (Fig. 3A). As expected, mRNA and protein levels of LRRC8A were significantly elevated in the OE group, which were 3.55 ± 0.72 (n = 4, *P* < 0.01, Figs. 3B) and 2.67 ± 0.30 folds (n = 3, *P* < 0.05, Fig. 3C and D) respectively for those in the lentivirus control group. Besides, an additional band, the uppermost one, was detected in the lane of the OE LRRC8A group, which was not referred to in the LRRC8A antibody specification. Thus, Sodium dodecyl sulfate-polyacrylamide gel electrophoresis (SDS-PAGE) gel of the corresponding location was cut and inspected by mass spectrometry. LRRC8A peptide was captured and detected successfully, suggesting that the band was LRRC8A (Fig. 3E). As illustrated in Fig. 3F, cells with over-expressed LRRC8A showed increased resistance to oxaliplatin at concentrations of 32 μM and 64 μM, respectively (n = 3, *P* < 0.05). For cells with over-expressed LRRC8A, the theoretically calculated IC_50_ of oxaliplatin was 80.66 μM, much higher than that for the control lentivirus-infected cells (37.20 μM).

**Fig. 3:**
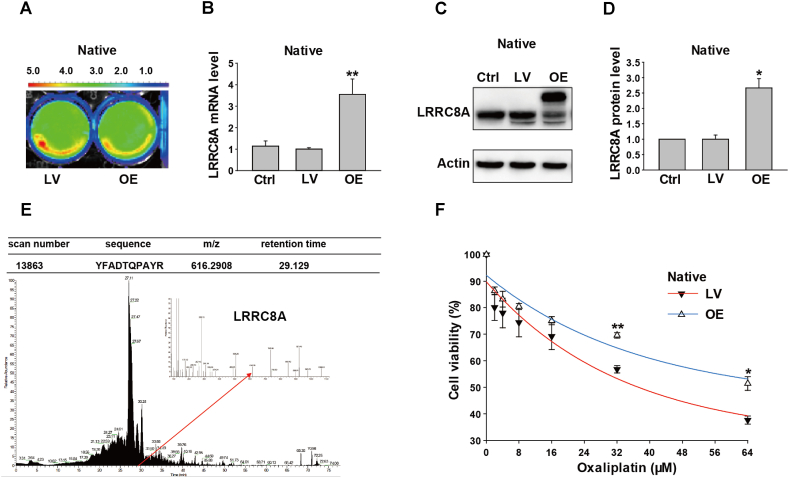
Effects of overexpression of LRRC8A on oxaliplatin sensitivities in the native HCT116 cells. (A) Images of HCT116 cells infected with the scramble or LRRC8A lentivirus vector were captured by the molecular imaging system. (B) The mRNA levels were assayed by qPCR and the transcriptional level of LRRC8A increased after cells were infected with the over-expressing LRRC8A lentivirus (OE) for 3 days. (C and D) The LRRC8A protein expressions were detected by Western blot, which was elevated by the over-expressing LRRC8A lentivirus (Supplementary Fig. 3). (E) The uppermost band in the OE lane was identified by mass spectroscopy and the unique peptide of LRRC8A was captured. (F) The over-expressed LRRC8A could enhance the resistance of native HCT116 cells to oxaliplatin. n = 3–4; *P < 0.05, **P < 0.01, ***P < 0.001.

Next, LRRC8A expression was downregulated by the specific LRRC8A short hairpin RNA (shRNA), of which the vector was packaged in lentivirus. As shown in Fig. 4A, the cells were successfully infected by LRRC8A shRNA lentivirus. And, LRRC8A mRNA and protein levels in the KD group were reduced to 57% (n = 3, *P* < 0.001, Fig. 4B) and 65% respectively for those in the control lentivirus group (n = 3, *P* < 0.05, Fig. 4C). After treatment with different concentrations of oxaliplatin for 24 h, cell viabilities in the NC group were significantly higher than those in KD group without exception (n = 3, *P* < 0.05, Fig. 4D). Therefore, these results suggested that LRRC8A may contribute to the sensitivity of colon cancer cells to oxaliplatin.

**Fig. 4:**
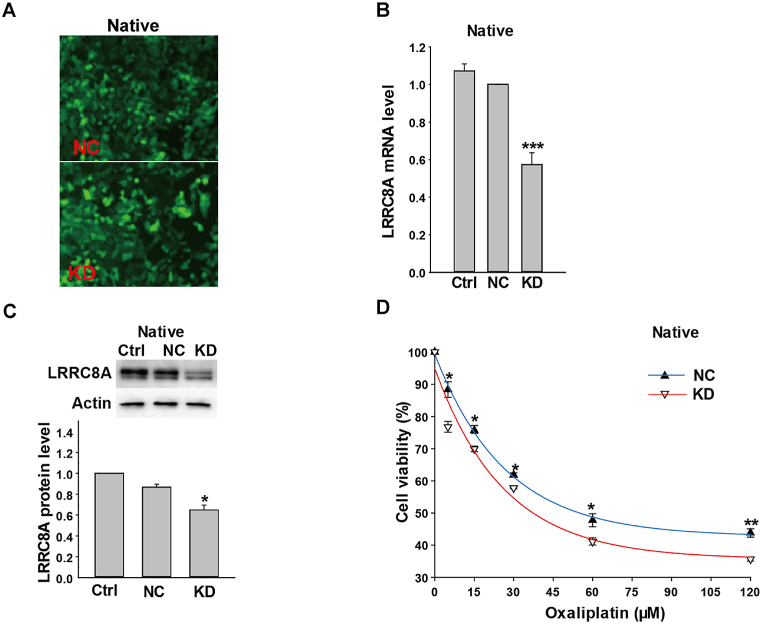
Knockdown LRRC8A decreased the resistance to oxaliplatin in native HCT116 cells. (A) Images of HCT116 cells infected with the scramble lentivirus or lentivirus packaging LRRC8A shRNA vectors, which expressed the green fluorescent protein. (B and C) The mRNA and protein expressions of LRRC8A were decreased in HCT116 cells infected with LRRC8A shRNA lentivirus in 3 days (Supplementary Fig. 4). (D) The down-regulation of LRRC8A proteins decreased the oxaliplatin resistance in the native HCT116 cells. n = 3; *P < 0.05, **P < 0.01, ***P < 0.001. (For interpretation of the references to color in this figure legend, the reader is referred to the Web version of this article.)

### 3.4 LRRC8A proteins were not essential in the maintenance of oxaliplatin-resistance in R-Oxa cells

The above results indicated that there was no significant difference in oxaliplatin resistance between R-Oxa and R-Oxa^dep^ cells, but the expression of LRRC8A in R-Oxa^dep^ cells was lower than that in R-Oxa cells. These suggested that LRRC8A maybe not be a vital factor in the maintenance of oxaliplatin resistance for R-Oxa cells.

We next measured mRNA and protein expression of LRRC8A by qPCR and Western blot after R-Oxa cells were infected with LRRC8A shRNA lentivirus for 72 h. The levels of LRRC8A mRNA and protein in the KD group were reduced to 55% (n = 4, *P* < 0.01, Fig. 5A) and 73% (n = 4, *P* < 0.05, Fig. 5B) respectively for those in the lentivirus control group. However, compared to the NC group, the sensitivity to oxaliplatin in the KD group was not changed (n = 3, *P* > 0.05, Fig. 5C). It is confirmed that LRRC8A was not essential for R-Oxa cells to maintain the resistance of oxaliplatin.

**Fig. 5:**
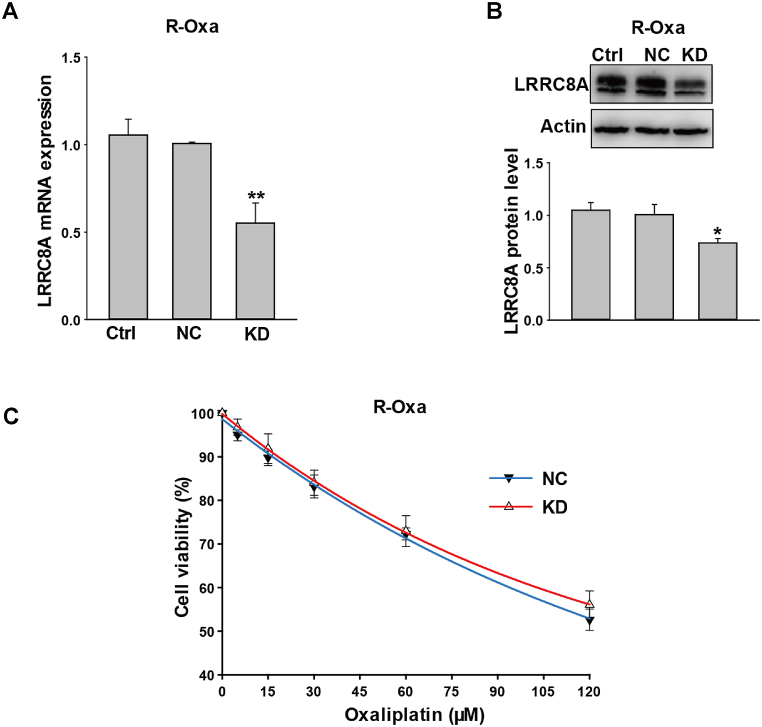
Down-regulation of LRRC8A expression had no influence on the drug resistance to oxaliplatin in R-Oxa cells. (A and B) The mRNA and protein expressions of LRRC8A were decreased after R-Oxa HCT116 cells were infected with LRRC8A shRNA lentivirus for 3 days (Supplementary Fig. 5). (C) Knocking-down LRRC8A expression in R-Oxa cells could not change the drug resistance to oxaliplatin. The cell viability was determined after treatment with different concentrations of oxaliplatin (5, 15, 30, 60, 120 μM) for 24 h. n = 3–4; *P < 0.05, **P < 0.01, ***P < 0.001.

### 3.5 Activation of platinum drug resistance pathway contributed to the maintenance of oxaliplatin resistance in colon cancer cells

**Fig. 6:**
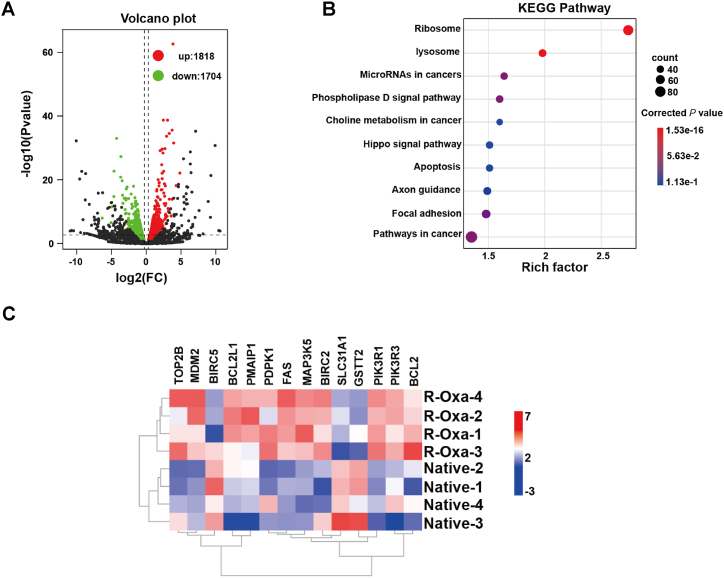
Gradual exposure to oxaliplatin-induced changes in the transcriptome profile of colon cancer cells. (A) Volcano map of differentially expressed genes between R-Oxa and native HCT116 cells (Red, upregulated genes; green, downregulated genes). (B) The top ten KEGG pathways enriched with the DEGs between R-Oxa and native HCT116 cells. (C) The expression heatmap of 14 DEGs involved in the platinum drug resistance pathway. n = 4. (For interpretation of the references to color in this figure legend, the reader is referred to the Web version of this article.)

The possible mechanisms in the maintenance of oxaliplatin resistance for R-Oxa cells were further explored via transcriptome sequencing. The cDNA libraries of native and R-Oxa HCT116 cells were constructed and sequenced, respectively. Compared to parental cells, 3522 DEGs were captured in R-Oxa cells, including 1818 up-regulated genes and 1704 down-regulated genes (n = 4, Fig. 6A). 2227 DEGs were successfully annotated in the KEGG pathway. In the top ten clustered pathways, the corrected *P* values for the ribosome and lysosome pathways were less than 0.05 (n = 4, Fig. 6B). Compared to the native HCT116 cells, there were 14 DEGs annotated in the pathway of platinum drug resistance, including the 3 down-regulated genes and the 11 up-regulated genes. The down-regulated genes solute carrier family 31 member 1 (SLC31A1), glutathione S-transferase theta 2 (GSTT2), and baculoviral IAP repeat containing 5 (BIRC5) were involved in drug uptake, drug inactivated and apoptosis inhibition, respectively. In the 11 up-regulated genes, except that DNA topoisomerase II beta (TOP2B) took part in nucleotide excision repair, and phorbol-12-myristate-13-acetate-induced protein 1 (PMAIP1) promoted apoptosis and other genes inhibited apoptosis (Fig. 6C). These suggested that there was a complex network to maintain the oxaliplatin resistance of R-Oxa cells, which refer to a variety of function-related factors.

## 4 Discussion

Oxaliplatin is used for the adjuvant chemotherapy of colon cancer after the surgical section. Unfortunately, clinical data indicated that about 50% of patients exhibited resistance to oxaliplatin with the extension of therapy time, which leads to treatment failure [17]. Thus, overcoming chemotherapy resistance is crucial for prolonging the survival time of cancer patients.

VRAC was first described in 1980 and its classical function is volume regulation by medicating Cl^−^ efflux when intracellular ion strength decreases [18]. A convincing review had classified that ion channels played an essential role in the development of drug resistance in cancer cells and provided evidence of VRAC's involvement [19]. A study found that suppressing VRAC activity with VRAC inhibitor NS3728 promoted the resistance of Ehrlich ascites tumor cells (EATC) to cisplatin [20]. But the inhibitor's lack of specificity and indistinct molecular determinant for VRAC obstructed the function research of VRAC. Later in 2014, two studies simultaneously reported that VRAC is composed of leucine-rich repeat-containing 8 heteromers and LRRC8A is a core component [8,9], that provides us a direct method to explore the function of VRAC. In the present study, we established an oxaliplatin-resistant HCT116 cell line by the oxaliplatin-induced method. Previous studies reported that the drug-resistant cell lines may partially forfeit their resistance after withdrawing the treatment of corresponding drugs [16,21,22]. While R-Oxa cells deprived of treatment of oxaliplatin for over 6 months still maintain a similar resistance characteristic as previously. It suggested that the R-Oxa cells established by us possessed stable resistance to oxaliplatin. Next, we observed that the LRRC8A expression in R-Oxa cells was significantly higher than that in native HCT116 cells, suggesting that LRRC8A may be an important factor in the acquisition of oxaliplatin resistance for colon cancer cells. Therefore, the effect of LRRC8A on oxaliplatin resistance in colon cancer cells was further investigated.

In our study, overexpression or knockdown of LRRC8A expression could alter the oxaliplatin resistance in native HCT116 cells, which suggest that LRRC8A contribute to the obtainment of oxaliplatin resistance in colon cancer cells. However, Knockdown LRRC8A expression in R-Oxa cells did not decrease the oxaliplatin resistance. Furthermore, R-Oxa^dep^ cells had a slight decrease in LRRC8A expression but conferred a similar oxaliplatin resistance characteristic to R-Oxa cells. The above results suggested that LRRC8A promotes the acquisition of oxaliplatin resistance but is not essential for its maintenance in colon cancer cells. Currently, the role of LRRC8A in drug resistance is not consistent in different cancer cell lines. For instance, overexpression of LRRC8A is responsible for decreased temozolomide resistance in U87/R cells [14] and knockdown of LRRC8A could promote the resistance to Pt-based anti-cancer drugs [13]. Conversely, the downregulation of LRRC8A increased the sensitivity of glioblastoma to temozolomide and carmustine [15]. These controversial conclusions may result from LRRC8A being associated with different LRRC8 family members to form tissue-specific VRACs. While different VRACs possess the specific property. For example, VRAC being composed of LRRC8A and LRRC8D was responsible for the 50% cisplatin uptake under isotonic conditions, but neither LRRC8C nor LRRC8E [13]. Thus, the mechanisms by which LRRC8A promotes the acquisition of oxaliplatin resistance in colon cancer cells need to be further explored.

The emergence of drug resistance in cancer cells may be referred to in multiple aspects, including drug accumulation, epigenetic alterations, the involvement of immune cells, tumor microenvironment (TME), epithelial-to-mesenchymal transition (EMT), DNA repair, apoptosis inhibition, and so on [[23], [24], [25], [26], [27]]. Transcriptome profiling analysis indicated that 3522 differentially expressed genes were found between native and R-Oxa cells and were significantly enriched in the ribosome and lysosome pathways. Further analysis indicated that 14 genes referred to drug uptake, drug inactivation, regulation of cell apoptosis, and nucleotide excision repair which belong to the platinum drug resistance pathway were significantly changed. Among them, SLC31A1, also named copper transporter 1 (CTR1), a major transporter of platinum drugs, is responsible for drug uptake [28]. In the present study, the reduced expression of CTR1 could decrease the oxaliplatin accumulation in R-Oxa cells. 9 of 11 upregulated genes in R-Oxa cells inhibited apoptosis progression, which favors the acquisition of drug resistance in colon cancer cells. Furthermore, the upregulated TOP2B was beneficial to DNA damage repair [29], which contributes to maintaining the oxaliplatin resistance of R-Oxa cells. Transcriptional regulation of genes in the platinum drug resistance pathway provided us direct evidence to explain how R-Oxa cells maintain resistance to oxaliplatin. It demonstrated that there was a complex network to maintain the oxaliplatin resistance of R-Oxa cells, which referred to a variety of function-related factors.

Although the present study demonstrates that LRRC8A could promote the development of oxaliplatin resistance, it is still not clear that the mechanisms of LRRC8A assisting colon cancer cells to acquire drug resistance. Furthermore, it remains elusive that the difference of LRRC8A expressions between the oxaliplatin-resistant and corresponding primary (non-oxaliplatin-resistant) colon cancer tissues. These are the future direction we will focus on, which would be helpful for elucidating the further mechanisms of LRRC8A in the promotion of oxaliplatin resistance.

In conclusion, LRRC8A is involved in the acquisition of oxaliplatin resistance rather than the maintenance in colon cancer cells. Transcriptional regulation of genes in the platinum drug resistance pathway may be an important mechanism to maintain oxaliplatin resistance in colon cancer cells.

## 5 Conclusions

LRRC8A promotes the initial development of oxaliplatin resistance in colon cancer cells. LRRC8A may be an intriguing potential target to interfere with the acquisition of oxaliplatin resistance for colon cancer cells in chemotherapy.

## Authors' contributions

Haifeng Zhang; Zhenghui Jing; Rong Liu: Performed the experiments; Analyzed and interpreted the data; Wrote the paper.

Yassin Shada; Sindhwani Shria; Shiyu Cui; Yuhua Ren: Performed the experiments; Analyzed and interpreted the data.

Yuan Wei: Performed the experiments; Analyzed and interpreted the data; Contributed reagents, materials, analysis tools or data.

Haifeng Zhang; Liangming Li; Shuang Peng: Conceived and designed the experiments; Contributed reagents, materials, analysis tools or data; Wrote the paper.

## Data availability

The data presented in this study are available on reasonable request from the corresponding author.

## Declaration of competing interest

The authors declare that they have no known competing financial interests or personal relationships that could have appeared to influence the work reported in this paper.

## Figures and Tables

**figs1:**
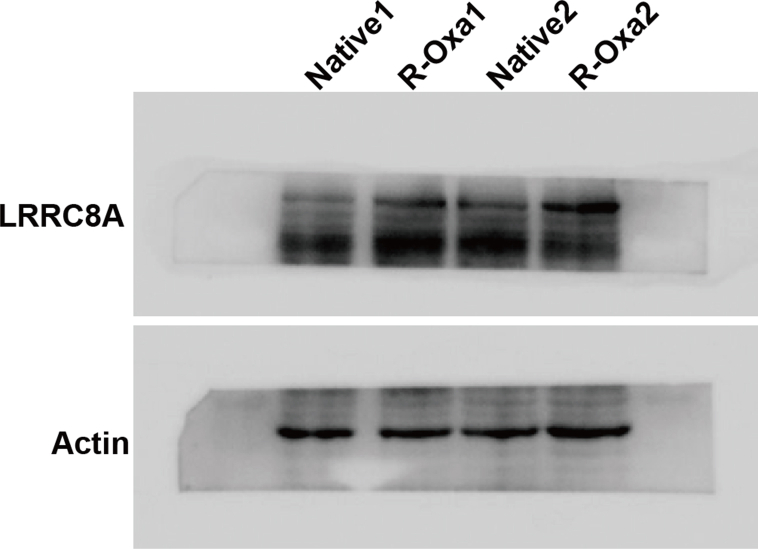


**figs2:**
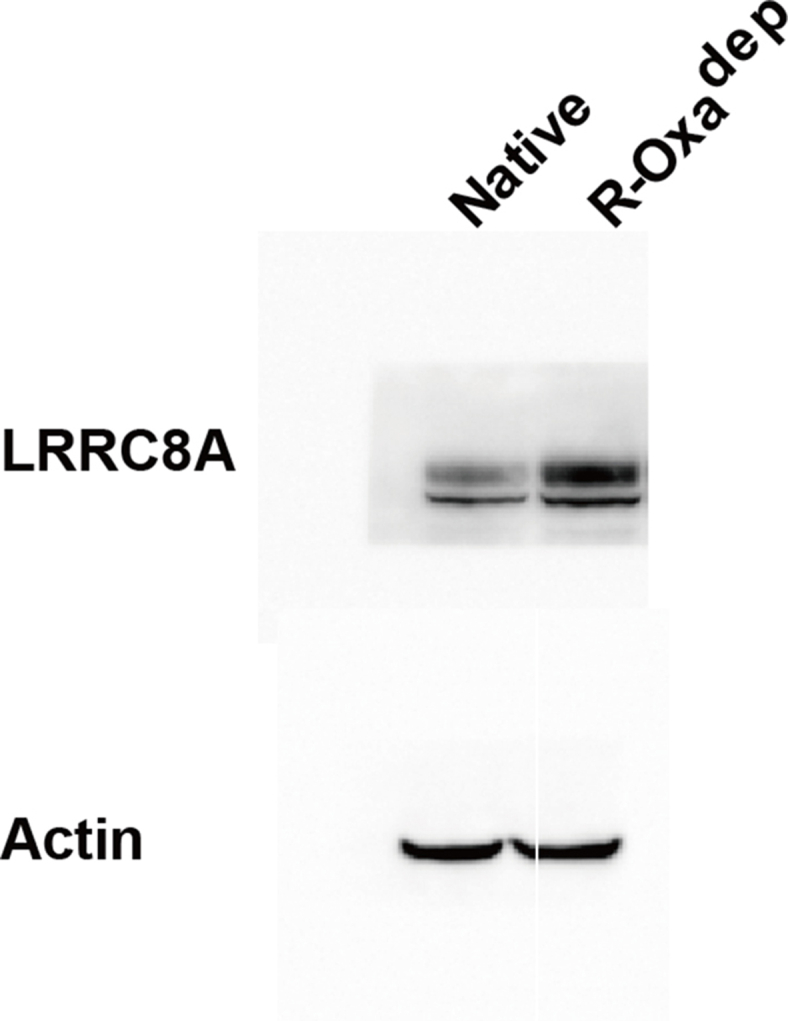


**figs3:**
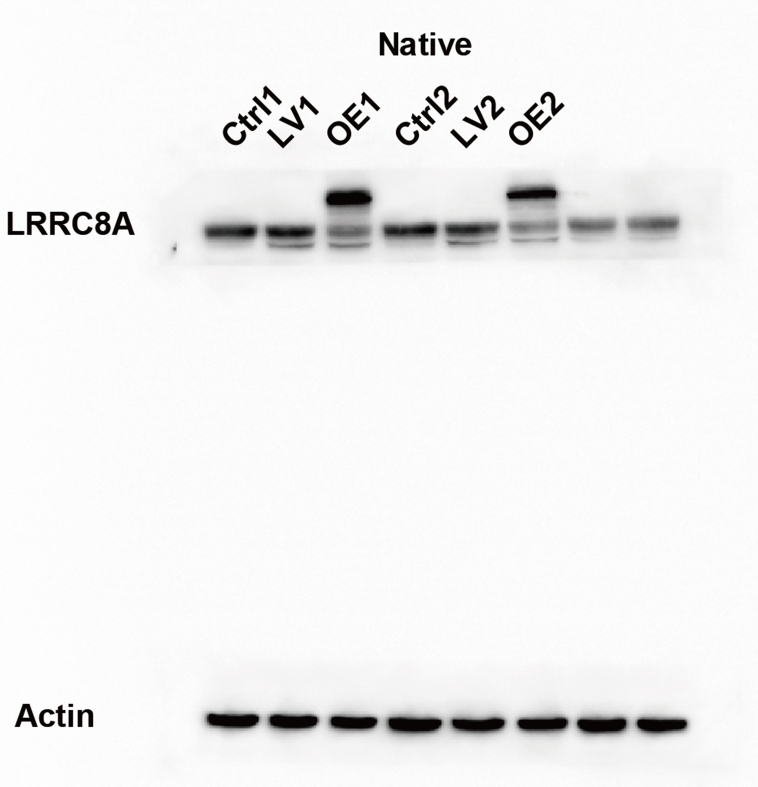


**figs4:**
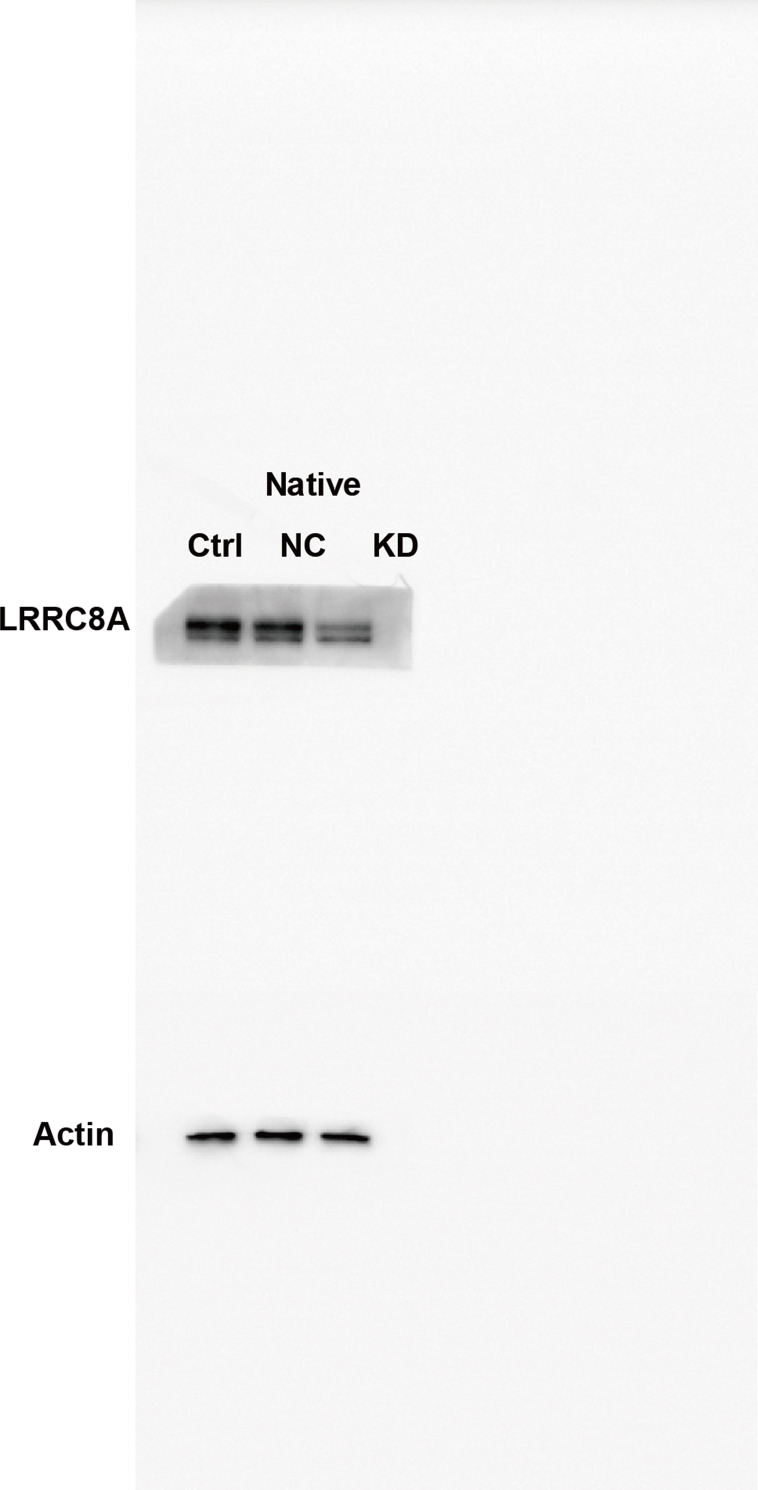


**figs5:**
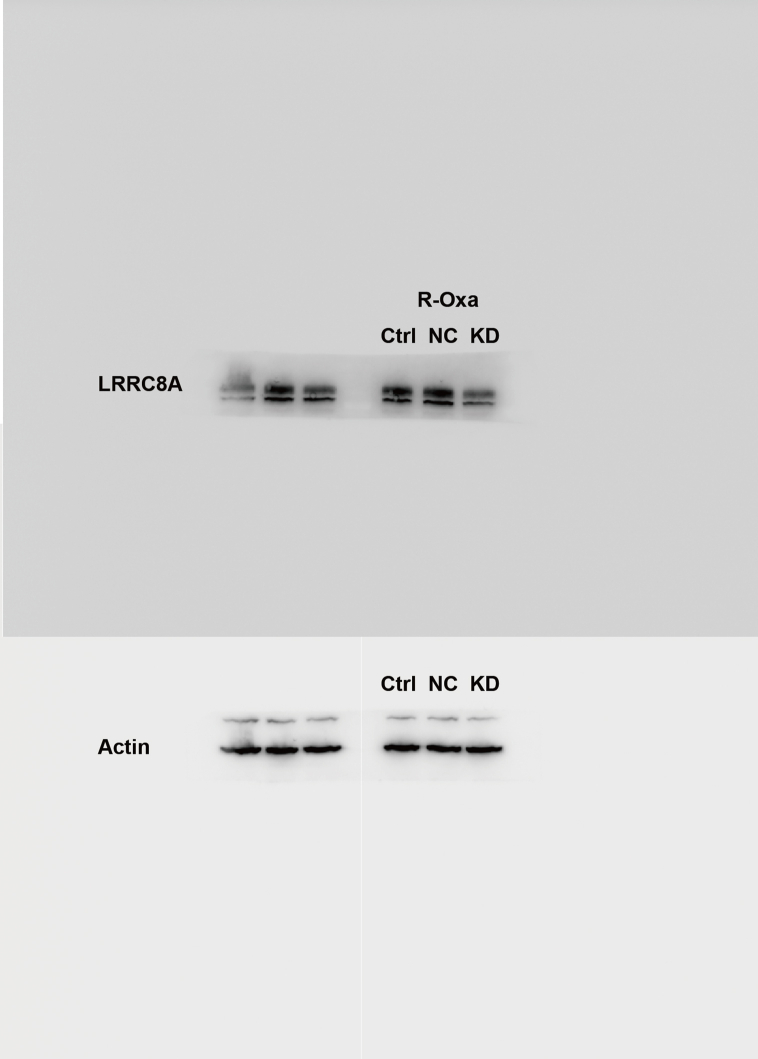


## Abbreviations

BCA: Bicinchoninic acid
BIRC5: Baculoviral IAP repeat containing 5
CCK-8: Cell counting kit-8
CRC: Colorectal cancer
CTR1: Copper transporter 1 CTR1
DEGs: Differentially expressed genes
GSTT2: Glutathione S-transferase theta 2
HRP: Horseradish peroxidase
IC_50_: Half maximal inhibitory concentration
IRAC: International agency for research on cancer
LRRC8A: Leucine-rich repeat-containing 8 A
PMAIP1: Phorbol-12-myristate-13-acetate-induced protein 1
PVDF: Polyvinylidene difluoride
RIPA buffer: Radioimmunoprecipitation assay buffer
R-Oxa: Oxaliplatin-resistant HCT116 cells
R-Oxa^dep^ cells: R-Oxa cells deprived of oxaliplatin treatment for over six months
RPMI 1640 medium: Roswell Park Memorial Institute 1640 medium
RT-qPCR: Reverse transcription quantitative polymerase chain reaction
SDS-PAGE: Sodium dodecyl sulfate polyacrylamide gel electrophoresis
shRNA: Short hairpin RNA
SLC31A1: Solute carrier family 31 member 1
STR: Short tandem repeat
