# Supplementary material for: LRRC8A promotes the initial development of oxaliplatin resistance in colon cancer cells
Source: Heliyon. 2023 Jun 1;9(6):e16872. doi: 10.1016/j.heliyon.2023.e16872 (PMC10258452; doi:10.1016/j.heliyon.2023.e16872)
Supplement: Multimedia component 1 [file mmc1.pdf]

# Cell Line Authentication Service

## STR Profile Report

| | |
|---|---|
| **Sample Submitted By:** | Dr. Zhaohui Chen<br>Shanghai Genechem Co., Ltd. |
| **Email Address:** | chenzh@genechem.com.cn |
| **Sales Order:** | 180727B |
| **Cell Line Designation:** | HCT 116 |
| **Date Sample Received:** | Jul 27th, 2018 |
| **Report Date:** | Jul 30th, 2018 |

**Methodology:** Nineteen short tandem repeat (STR) loci plus the gender determining locus, Amelogenin, were amplified using the commercially available EX20 Kit from AGCU. The cell line sample was processed using the ABI Prism® 3500 Genetic Analyzer. Data were analyzed using GeneMapper® ID-X v1.4 software (Applied Biosystems). Appropriate positive and negative controls were run and confirmed for each sample submitted.

**Data Interpretation:** Cell lines were authenticated using Short Tandem Repeat (STR) analysis as described in 2012 in ANSI Standard (ASN-0002) by the ATCC Standards Development Organization (SDO) and in Capes-Davis et al., Match criteria for human cell line authentication: Where do we draw the line? Int J Cancer. 2013;132(11):2510-9.

**GTB™ performs STR Profiling following ISO 9001:2008 and ISO/IEC 17025:2005 quality standards.** There are no warranties with respect to the services or results supplied, express or implied, including, without limitation, any implied warranty of merchantability or fitness for a particular purpose. Genetic Testing Biotechnology (GTB) is not liable for any damages or injuries resulting from receipt and/or improper, inappropriate, negligent or other wrongful use of the test results supplied, and/or from misidentification, misrepresentation, or lack of accuracy of those results. Your exclusive remedy against GTB and those supplying materials used in the services for any losses or damage of any kind whatsoever, whether in contract, tort, or otherwise, shall be, at GTB's option, refund of the fee paid for such service or repeat of the service.

The GTB™ is a registered trademark of Genetic Testing Biotechnology Corporation (Suzhou).

Technical Questions?
GTB Technical Support
+86-512-62806339
service@jsdna.org
Section 303, Yixin BLD
SIP, Suzhou, 215123
Jiangsu, P.R. China

Ordering Questions?
order@jsdna.org
GTB Corporation
+86-512-62806339
Section 303, Yixin BLD
SIP, Suzhou, 215123
Jiangsu, P.R. China

GENETIC TESTING BIOTECHNOLOGY

# Cell Line Authentication Service

## STR Profile Report

**Sales Order:** 180727B

| | Test Results for Submitted Sample | | | DSMZ Reference Database Profile | |
|---|---|---|---|---|---|
| **Loci** | **Query Profile:** HCT 116 | | | **Database Profile:** HCT 116 | |
| Amelogenin | X | Y | | X | Y |
| D3S1358 | 12 | 18 | 19 | | |
| D13S317 | 10 | 12 | | 10 | 12 |
| D7S820 | 11 | 12 | | 11 | 12 |
| D16S539 | 11 | 13 | | 11 | 13 |
| Penta E | 13 | 14 | | | |
| TPOX | 8 | 9 | | 8 | 9 |
| TH01 | 8 | 9 | | 8 | 9 |
| D2S1338 | 16 | | | | |
| CSF1PO | 7 | 10 | | 7 | 10 |
| Penta D | 9 | 13 | | | |
| D19S433 | 12 | 13 | | | |
| vWA | 17 | 22 | | 17 | 22 |
| D21S11 | 29 | 30 | | | |
| D18S51 | 16 | 17 | | | |
| D6S1043 | 13 | 14 | | | |
| D8S1179 | 11 | 12 | 14 | | |
| D5S818 | 10 | 11 | | 10 | 11 |
| D12S391 | 17 | 21 | 22 | | |
| FGA | 18 | 23 | | | |

*The allele match algorithm compares the 8 core loci plus amelogenin only, even though alleles from all loci will be reported when available.*

*Note: Loci highlighted in grey (8 core STR loci plus Amelogenin) can be made public to verify cell identity. In order to protect the identity of the donor,* ***please do not publish*** *the allele calls from all the STR loci tested.*

### Explanation of Test Results

Cell lines with ≥80% match are considered to be related; i.e., derived from a common ancestry. Cell lines with between a 55% to 80% match require further profiling for authentication of relatedness.

- [ ] The submitted sample profile is human, but not a match for any profile in the DSMZ STR database.
- [x] The submitted profile is an exact match for the following human cell line(s) in the DSMZ STR database (8 core loci plus Amelogenin): HCT 116
- [ ] The submitted profile is similar to the following DSMZ human cell line(s):

e-Signature, Technician: Digitally signed by Faye Wong
DN: cn=Faye Wong, o=Genetic Testing Biotechnology (Suzhou), ou=DNA Typing Section, email=order@jsdna.org, c=CN
Date: 2018.07.30 16:56:31 +08'00'

GENETIC TESTING BIOTECHNOLOGY · GUARANTEE · CELL LINE AUTHENTICATION

e-Signature, Reviewer: Digitally signed by Alan Cui
DN: cn=Alan Cui, o=Genetic Testing Biotechnology (Suzhou), ou=Supervision Section, email=service@jsdna.org, c=CN
Date: 2018.07.30 16:56:55 +08'00'

More information

**Addendum:** Electropherogram/matching results for the customer’s sample set 1 of 1

GENETIC TESTING BIOTECHNOLOGY

# Cell Line Authentication Service

## STR Profile Report

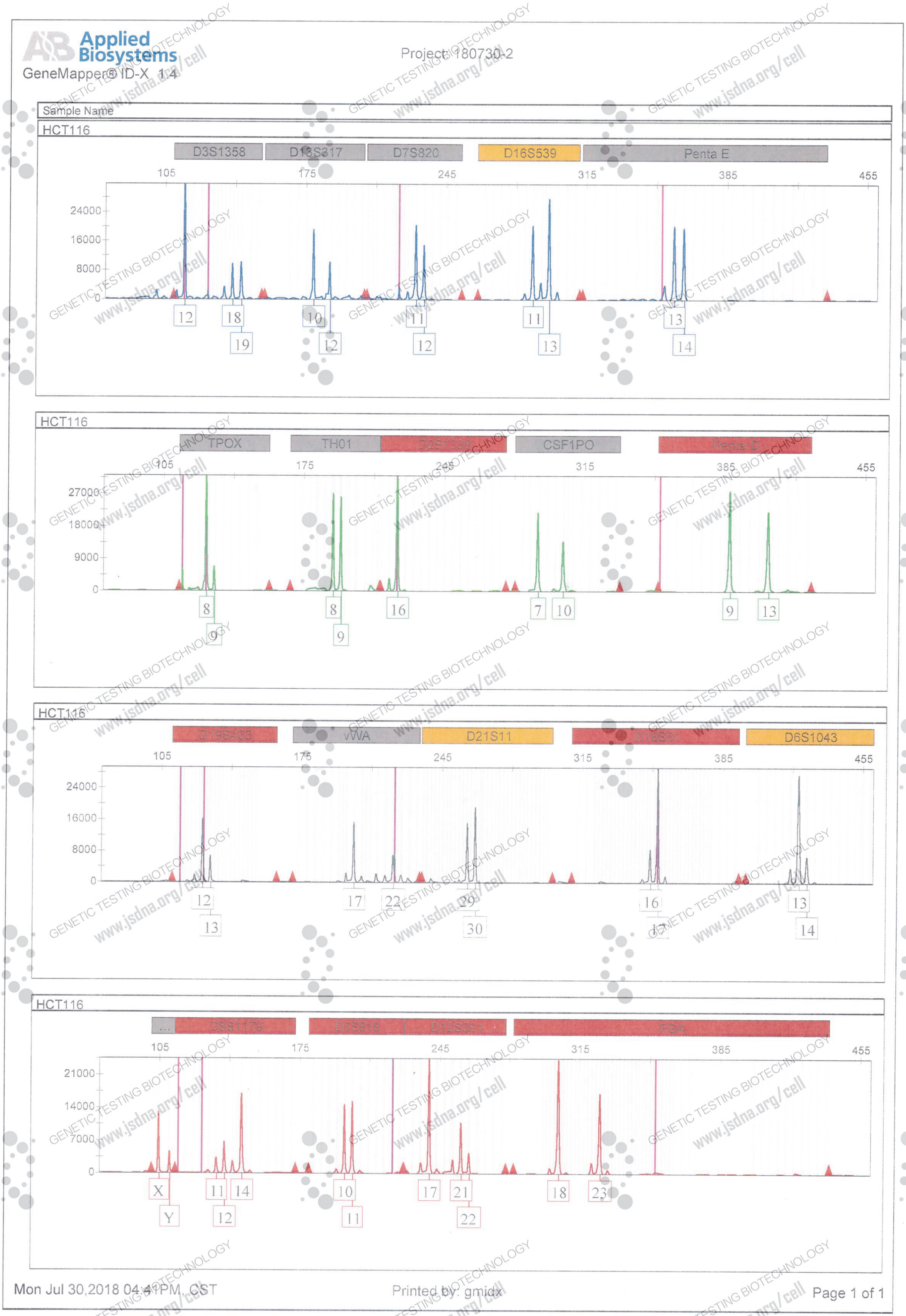


GENETIC TESTING BIOTECHNOLOGY www.jsdna.org/cell
